# Clinical advantages and neuroprotective effects of monitor guided fang’s capillary fascia preservation right RLN dissection technique

**DOI:** 10.3389/fendo.2022.918741

**Published:** 2022-07-22

**Authors:** Qian Shi, Jiaqi Xu, Jugao Fang, Qi Zhong, Xiao Chen, Lizhen Hou, Hongzhi Ma, Lin Feng, Shizhi He, Meng Lian, Ru Wang

**Affiliations:** Department of Otorhinolaryngology Head and Neck Surgery, Beijing Tongren Hospital, Capital Medical University, Beijing, China

**Keywords:** thyroid cancer, recurrent laryngeal nerve, neuroprotective, lymph node dissection, parathyroid preservation

## Abstract

**Objective:**

To investigate the feasibility and advantages of Fang’s capillary fascia preservation right recurrent laryngeal nerve (RLN) dissection technique (F-R-RLN dissection) with preservation of the capillary network and fascia between the RLN and common carotid artery for greater neuroprotective efficiency compared with traditional techniques.

**Methods:**

We retrospectively analyzed 102 patients with papillary thyroid carcinoma undergoing right level VI lymph node dissection in our department from March 2021 to January 2022. Sixty patients underwent F-R-RLN dissection (the experimental group) and 42 patients underwent standard dissection (the control group). The intraoperative electrical signal amplitude ratios of the RLN, the number of dissected lymph nodes, and the preservation rates of the parathyroid glands were recorded and compared between the two groups.

**Results:**

The electrical signal amplitude ratio of the lower neck part point of the RLN to the upper laryngeal inlet point in the experimental group was significantly lower than the ratio in the control group (*p* = 0.006, Z-score = -2.726). One patient suffered transient RLN paralysis in both groups, but this resolved within 1 month after operation. There were no significant differences between the two groups in terms of the number of level VIa or level VIb lymph nodes dissected, nor in the rate of preservation of the parathyroid glands.

**Conclusions:**

F-R-RLN dissection is a thorough dissection technique that is effective at preventing an electrical signal amplitude decrease in the RLN, and at preventing RLN paralysis by preserving its blood supply.

## Introduction

Papillary thyroid carcinoma accounts for more than 90% of malignant thyroid tumors, and its incidence has been increasing over recent decades (1). In spite of its indolent characteristics, it has a propensity to spread to the lymph nodes, particularly those in the level VI compartment (2). Due to the potential injury to the right recurrent laryngeal nerve (RLN) (3), the best approach to level VI lymph node dissection remains a debated issue. In traditional lymph node dissection, the middle and lower parts of the right cervical RLN are separated from surrounding tissues to achieve adequate dissection of the lymph nodes posterior to the nerve, which may cause interruption or even devascularization of the RLN. Since March 2021, Monitor Guided Fang’s Capillary Fascia Preservation Right RLN Dissection Technique (F-R-RLN dissection) has been adopted by our department. In this technique, the RLN is slightly spared laterally to facilitate both exposure of the dissection area and preservation of the capillary network adjacent to the RLN. This study aimed to investigate the feasibility and advantages of F-R-RLN dissection compared with the traditional level VI dissection method, which involves dissecting the nerve circumferentially.

## Materials and methods

### Patients

A total of 102 patients with papillary thyroid carcinoma requiring right level VI lymph node dissection from March 2021 to January 2022 at the Department of Otolaryngology Head and Neck Surgery in Beijing Tongren Hospital were retrospectively included. The inclusion criteria were: (1) The invoked potential signal ratio R1/R2d (distal R2: the R2 in the upper laryngeal inlet part) was between 90 to 110%, which suggested the intubation was correct, (2) the nerve monitoring circuit was integral, and (3) they were not disturbed by muscle relaxants. The exclusion criteria were: (1) confirmed invasion (established preoperatively or intraoperatively) to the RLN or tracheoesophagus from the primary tumor or the lymph nodes; (2) history of vocal cord dysfunction; or (3) cases of papillary thyroid carcinoma relapse. The study was approved by the ethics review board of the Beijing Tongren Hospital, Capital Medical University, and all patients signed informed consent.

Sixty patients underwent F-R-RLN dissection and 42 patients underwent traditional lymph node dissection. All patients were treated by the same group of healthcare practitioners, which included anesthetists, surgeons, and laryngoscopy operators. Intraoperative neuromonitoring (IONM) was conducted using the Medtronic Xomed NIM-Response 3.0, with the electric current set to 2.0 mA. Surgery was performed under general endotracheal anesthesia. An injection of 0.2 mg/kg of Mivacron (muscle relaxant with effective maintenance time of about 20 minutes) was used for induction of anesthesia and no additional muscle relaxant and antagonist were administrated during surgery. We began to measure R1 30-40 minutes after the induction of anesthesia to reduce the disturbance of muscle relaxant. In all patients, electrical signals from the vagus nerve and RLN were routinely measured using the standard four-step (V1-R1-R2-V2) IONM procedure (4, 5).

### Surgical technique

**Figure 1:**
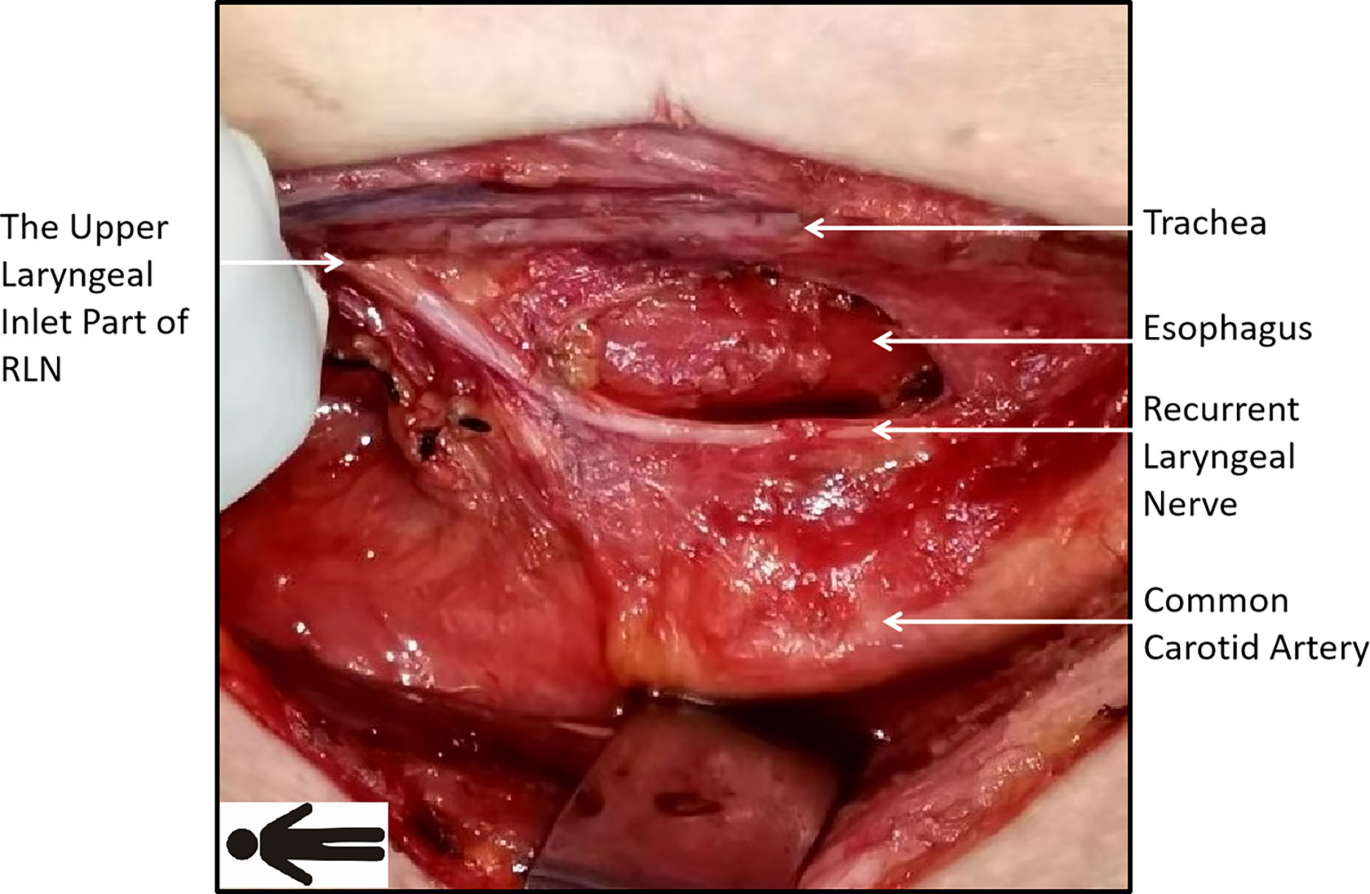
Course of the right recurrent laryngeal nerve (RLN): the right RLN runs obliquely with its inferior side closer to the common carotid artery. Level VIb, limited by the nerve and trachea, is shaped as a triangle with its top on the upper side.

Level VIb is primarily located in the area delineated by the RLN and trachea. It has a triangular shape due to the anatomical characteristics of the right RLN as it bends around the subclavian artery, and the caudal side is adjacent to the common carotid artery (**Figure 1**). Based on these anatomical features, F-R-RLN dissection highlights that an adequate exposure and dissection of the level VIb lymph nodes can be achieved by slightly dislocating the RLN laterally to the surface of the common carotid artery and dislocating the laryngeal body to the left and upper side.

#### F-R-RLN dissection for the experimental group

All surgeries commenced with routine incision of the skin and subcutaneous tissue and freeing of the strap muscles to expose the right lobe of the thyroid and right level VI lymphatic connective tissue. Next, the central lymphatic fatty tissue superior to the RLN was dissected *en bloc*; this included the perithyroid, pretracheal, and tracheoesophageal groove lymph nodes. The right level VIa dissection began from the level of the hyoid bone and continued down to the level of the suprasternal notch or the upper edge of the innominate artery. The area extended laterally to the right common carotid artery and medially to the paratracheal lymph nodes and was limited to the level of the RLN. The right lymphatic fatty tissue inferior to the RLN was identified and excised in an ipsilateral level VIb dissection. In VIb dissection, the medial border was the trachea and the lateral border was the inner edge of the common carotid artery. The upper border was the entry site of the RLN into the larynx and the lower border was the intersection of the innominate artery and tracheoesophageal groove. The level VIb dissection was posteriorly limited at the level of the esophageal wall and superficially limited at the level of the RLN.

**Figure 2:**
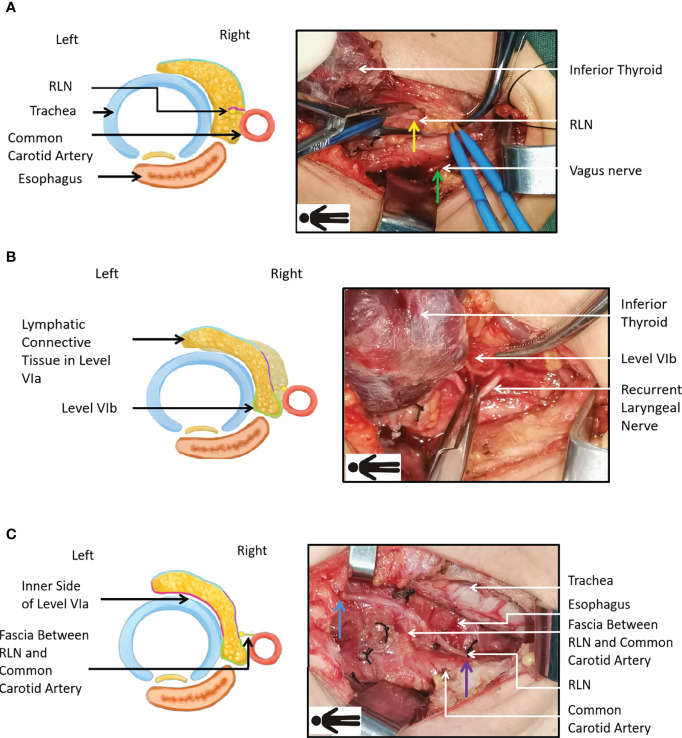
Technique of Fang’s capillary fascia preservation right recurrent laryngeal nerve (F-R-RLN) dissection. **(A)** Dissecting superficial lymph connective tissue of RLN and cutting the lateral border of level VIa (the purple line in the figure). The green arrow points to the V1 electrical signal monitor point from the vagus nerve and the yellow arrow points to the R1 electric signal monitor point from the RLN. **(B)** Sparing RLN laterally to the superior and inner side of the common carotid artery and dissecting level VIb lymph connective tissue (the green line in the figure). **(C)** Preserving the capillary network encased by the fascia between the RLN and the common carotid artery and dissecting level VI lymph connective tissue (the red line in the figure). The blue arrow points to the R2d in the upper laryngeal inlet part. The purple arrow points to the R2p in the lower neck part. *Laryngeal body is dislocated to the left and upper side in order to expose level VIb and therefore the esophagus is shifted to the right side of the trachea.*.

Electrical signals from the vagus nerve were recorded as V1. The vagus nerve was inspected and exposed, the middle thyroid vein was severed, and the ipsilateral thyroid lobe was raised to the medial side. The inferior parathyroid gland and its vascular supply were identified. The thyrothymic ligament was raised in a fan shape, such that the vertex was formed by the inferior parathyroid gland, while preserving the vascular pedicle of the parathyroid gland. Next, the inferior parathyroid gland and its vascular supply were freed to the lateral inferior side. A probe was inserted at the RLN in the tracheoesophageal groove at the level of the inferior thyroid pole and R1 was recorded (**Figure 2A**). The lymphatic connective tissue superficial to the RLN was briefly inspected, after which the lateral border of the level VIa area was incised and the area from the entrance of the inferior thyroid artery into the thyroid to the caudal site of the common carotid artery was dissected. At the caudal pole of the common carotid artery, the RLN was commonly adherent to the artery with less anatomical variation, which formed a suitable starting point for RLN dissection. The RLN was gently dislocated to the medial edge of the common carotid artery while dislocating the larynx to the upper left for adequate exposure of the right level VIb compartment. A meticulous level VIb dissection was performed along the esophagus after removing lymphatic fatty tissue. These tissue samples were preserved (**Figure 2B**). Branches of the RLN were severed by surgical scissors, which minimized potential electrical injury to the stem by electric scalpels. A layer of thin fascia encasing the capillaries of the RLN was then exposed between the RLN and common carotid artery. The medial border of the level VIa lymphatic connective tissue was separated along the tracheal midline after dissection from the lateral wall of the trachea to its midline (**Figure 2C**). The inferior limit was cut off at the level of the suprasternal fossa. The prelaryngeal lymph nodes were then meticulously dissected. The RLN was dissected upwards to its entrance into the larynx, after which the thyroid lobe was completely excised. The evoked potential R2 in the lower neck part was defined as proximal R2 (R2p), and the evoked potential R2 in the upper laryngeal inlet part was defined as distal R2 (R2d) (**Figure 2C**). Additional tissue was excised based on the tumor location and size (**Table 1**).

**Table 1:** The procedure during Fang’s capillary fascia preservation right recurrent laryngeal nerve (F-R-RLN) dissection technique.

Steps	Operation
Step 1	Initial vagal nerve stimulation (V1)
Step 2	RLN stimulation at the level of the inferior thyroid pole (R1)
Step 3	Incision of the lateral border of the level VIa area
**Step 4**	**Dislocation of the RLN to the medial edge of the common carotid artery**
Step 5	Dislocation of the larynx to the upper left for complete exposition of the VIb level
Step 6	Dissection of the VIb level along the esophagus
**Step 7**	**Preservation of the thin fascia encasing the capillaries of the RLN between the RLN and common carotid artery**
Step 8	Separation of the medial and inferior border of the level VIa along the trachea
Step 9	Dissection of the prelaryngeal lymph nodes
Step 10	Dissection of the RLN to its entrance into the larynx and excision of the thyroid lobe
Step 11	Stimulation of the RLN in the lower neck part (R2p) and the upper laryngeal inlet part (R2d), as well as stimulation of the vagal nerve (V2)

The bold words show the key steps in the procedure.

#### Conventional level VI dissection technique for the control group

In the control group, the middle and lower two-thirds of the right cervical RLN were separated from the surrounding tissue circumferentially and moved upwards for adequate dissection of the lymph nodes. Apart from the technique used for exposure of the level VI compartment, the remaining surgical steps were similar to those in the experimental group.

### Data recorded

Evoked potentials V1, R1, R2p, R2d, and V2 were recorded for all patients. R2p and R2d were recorded after level VI dissection. The regression of neural electromyography (EMG) signals after exposing the entire RLN was quantified by the ratio of R2p to R2d. Postoperative vocal cord movement was also evaluated by stroboscopy. The number of dissected lymph nodes *en bloc* in levels VIa and VIb, the number of metastasized lymph nodes according to pathological reports, the preservation rate of the parathyroid glands *in situ*, and the changes in serum calcium and parathyroid hormone before and after surgery were recorded. In our study, hypoparathyroidism was defined as serum intact parathyroid hormone (iPTH) < 15 ng/L (normal range: 15–65 ng/L) or postoperative clinical symptoms of hypocalcemia (neuromuscular irritability including paresthesia, muscle cramps, tetany, or seizures) with or without a serum calcium level of less than 2.0 mmol/L (8.0 mg/dL). Transient hypoparathyroidism was defined as postoperative hypoparathyroidism lasting less than 6 months. Permanent hypoparathyroidism was defined as postoperative hypoparathyroidism lasting more than 6 months (6).

### Statistical analysis

SPSS 20.0 (Chicago, Illinois, USA) was used for statistical analysis. Data on the number of dissected lymph nodes were presented as medians (quartiles) because of their non-normal distribution. Two independent sample t-tests or Mann-Whitney U tests were conducted to compare numeric variables. The differences in categorical variables were analyzed with the Pearson Chi-square test or Fisher’s exact test. P-values < 0.05 were labeled statistically significant.

## Results

**Figure 3:**
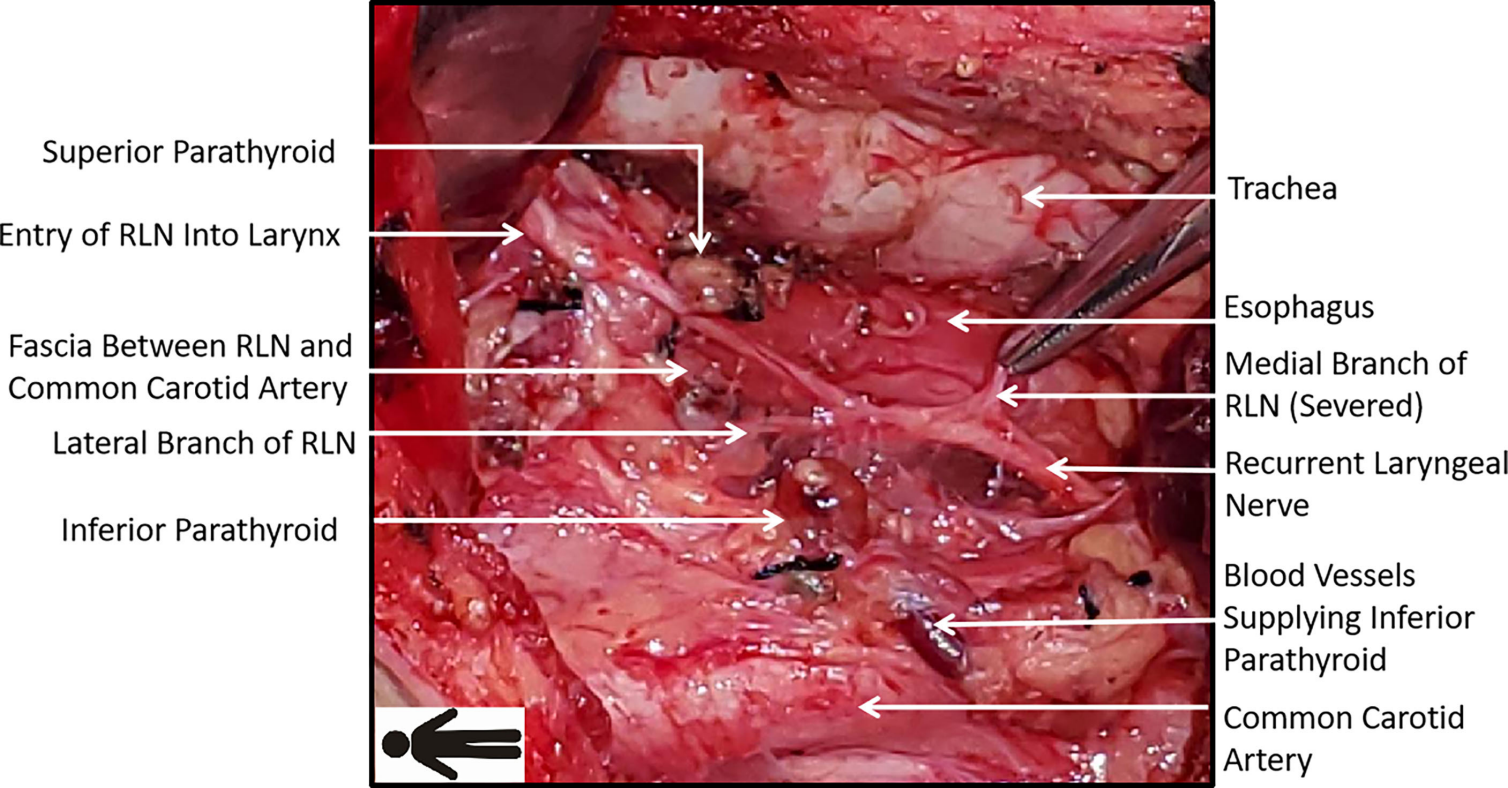
Operative field after Fang’s capillary fascia preservation right recurrent laryngeal nerve (F-R-RLN) dissection technique: the capillary network encased by fascia between the RLN and the common carotid artery and the branches of RLN running laterally are preserved.

In the F-R-RLN dissection, the RLN is mildly dislocated and only the medial branches are severed. The capillary network encased by fascia between the RLN and the common carotid artery is preserved. **Figure 3** shows the operative field after operation.

### Patients and disease characteristics

The baseline demographic and clinical characteristics of patients are shown in **Table 2**. There were no significant differences between the experimental and control groups in gender, the number of primary lesions, the size of primary lesions, T stage, N stage, invasion of strap muscles, concomitant or not with Hashimoto’s thyroiditis, and preferences for partial versus total thyroidectomy.

**Table 2:** Patients and disease characteristics between two groups.

	Experimental Group (n=60)	Control Group (n=42)	P-Value
Gender			0.670
Male	19	15	
Female	41	27	
Number of lesions			0.111
Single	32	29	
Multifocal	28	13	
The size of primary lesions*			0.254
<1cm	27	20	
1-2cm	21	8	
2-4cm	7	9	
>4cm	5	5	
N stage			0.650
N0	27	17	
N1*	33	25	
< 0.5cm	25	20	
0.5-1.0cm	5	3	
>1.0cm	3	2	
Hashimoto’s Thyroiditis			0.283
Yes	17	8	
No	43	34	
Invasion of strap muscles			1.000
Yes	3	2	
No	57	40	
Surgical Procedure			0.924
Right lobe and isthmus + right level VI dissection	28	20	
Total thyroidectomy + bilateral level VI dissection	32	22	

*The size of primary lesions was the greatest diameter of a primary lesion diagnosed by pathology (the greatest diameter if multifocal). The patients with N1 were stratified according to the maximum diameter of the invaded lymph nodes in the right level VI.

### Variations in EMG signal amplitude

The R2p/R2d ratio was categorized into three levels (>90%, 50%~90%, <50%), and patients in both the control and experimental groups were divided into these 3 subcategories. The cases of each subcategory, their percentages in the total cases, and the prevalence of vocal cord dysfunction in each subgroup were recorded (**Table 3**). There were significant differences between the control and experimental groups in terms of EMG amplitude changes (the non-parametric Mann-Whitney test, *p* = 0.006, Z-score = -2.726). In one patient in the experimental group, R2p and V2 were lost during surgery in spite of a complete RLN stem. The patient experienced hoarseness on the first postoperative day, and subsequent laryngoscopy showed that his right vocal cord was fixed at the paramedian position. His vocal cord movement and phonation recovered 1 month after surgery. The rate of transient RLN paralysis in the experimental group was 1.7%. In the control group, one patient also experienced signal loss of R2p and V2 in spite of an apparently intact RLN trunk. Similar to the patient in the experimental group, hoarseness and vocal cord fixation was observed on the first postoperative day, which resolved 1 month after surgery.

**Table 3:** Variation of recurrent laryngeal nerve (RLN) signal amplitude and vocal cord movement after level VI lymph node dissection.

	R2p/R2d	Experimental group			Control group		
		Case of RLN	Ratio (%)	Vocal cord dysfunction	Case of RLN	Ratio (%)	Vocal cord dysfunction
Group 1	>90%	38	63.3 (38/60)	0	15	35.7 (15/42)	0
Group 2	50%~90%	20	33.3 (20/60)	0	24	57.1 (24/42)	0
Group 3	<50%	2	3.3 (2/60)	1	3	7.1 (3/42)	1

R2p, proximal R2, RLN signal amplitude in the lower neck part after dissection; R2d, distal R2, RLN signal amplitude in the upper laryngeal inlet part after dissection.

### Number of dissected lymph nodes and metastasis rate

There were no significant differences between the experimental and control groups in the number of dissected lymph nodes and the metastasis rate. In the experimental group, the overall number of dissected level VI lymph nodes was significantly larger compared with the number in the level VIa area, which indicated that enough VIb lymph nodes were dissected in the experimental group and F-R-RLN dissection technique might help to dissect the level VI lymph nodes thoroughly (**Tables 4**, **5**).

**Table 4:** Dissected lymph nodes in level VI.

	Experimental group			Control group			P-value of Medians
	Q1	Median (Min-max)	Q3	Q1	Median (Min-max)	Q3	
lymph nodes in level VIa	4	6 (1-22)	8	3	4.5 (1-13)	6	0.513
lymph nodes in level VIb	1	2.5 (0-8)	4	1	3 (0-8)	3	0.699

Lymph nodes in level VIa and VIb were represented by their respective medians. Q1: the first quartile; Q3: the third quartile. Min: the minimal number of lymph nodes dissected in the level VIa and VIb groups; Max: the maximal number of lymph nodes dissected in the level VIa and VIb groups.

**Table 5:** Level VI lymph node metastasis ratio.

	Experimental group	Control group
level VIa metastasis	55.0% (33/60)	40.5% (17/42)
level VIb metastasis	15% (9/60)	11.9% (5/42)

Level VIa and VIb metastasis rates were calculated by the ratio of patients for whom lymph node metastasis occurred to the total number of patients in each group.

### Preservation of the right inferior parathyroid gland

**Figure 4:**
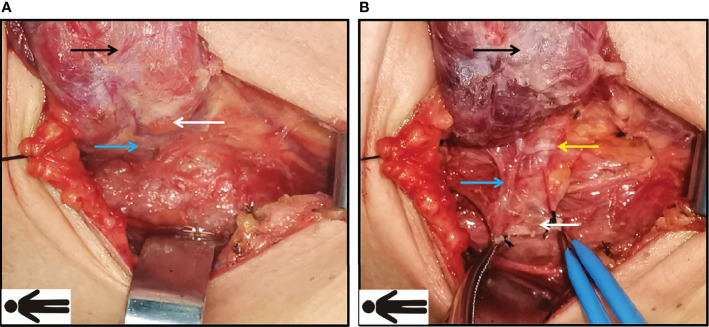
Preservation of inferior parathyroid gland during Fang’s capillary fascia preservation right recurrent laryngeal nerve (F-R-RLN) dissection technique. **(A)** Inspect the inferior parathyroid glands (the white arrow) and its blood supply (blue arrow) after raising the lobe. **(B)** Protect and raise sternothyroid ligament outwards and downwards, creating a fan-like shape, in which the vertex is formed by the parathyroid and the parathyroid blood vessels; the sternothyroid ligament is intact. *Black arrow: the right lobe of thyroid; white arrow: the inferior right parathyroid gland; blue arrow: the blood supply of parathyroid gland; yellow arrow: the recurrent laryngeal nerve (RLN)*.

In F-R-RLN dissection, the protection of the parathyroid gland is initiated prior to the dissection. The inferior parathyroid glands are initially inspected after raising the right thyroid lobe. If the parathyroids are clearly exposed and the blood supply can be identified, the sternothyroid ligament is moved outwards and downwards, creating a fan-like shape, in which the vertex is formed by the parathyroid glands and the pedicle is formed by the parathyroid blood vessels (**Figure 4**).

No significant differences were demonstrated between the two groups in terms of the *in-situ* preservation rates, implantation rates, or total detection rates of the right inferior parathyroid gland: these were 76.6% (46/60), 16.6% (10/60), and 93.3% (56/60), respectively, for the experimental group and 78.6% (33/42), 11.9% (5/42), and 90.6% (38/42), respectively, for the control group. For patients undergoing total thyroidectomy, postoperative transient hypoparathyroidism occurred in nine cases in the experimental group (28.1% or 9/32) and in five cases in the control group (22.7% or 5/22). There were no significant differences between the two groups. Permanent hypoparathyroidism was not reported in either group.

## Discussion

Papillary thyroid carcinoma has a predilection to metastasize to the cervical lymph nodes. One of the most common compartments of cervical lymph node metastasis is level VI (7), where the prevalence of metastasis can reach 47.6%, even in thyroid microcarcinoma (8). Additionally, occult metastasis to level VI lymph nodes can be detected by pathology in 50% of patients who have no radiological evidence of lymph node metastasis before surgery (9). It has been suggested that cervical central lymph node metastasis is closely associated with both local recurrence and with the occurrence of lymph node metastasis after initial cancer surgery (10). Postoperative recurrence and level VI lymph node metastases increase the likelihood of impacting the adjacent nerves and parathyroid glands during re-operation because of adhesion, indistinct anatomic structure, and scarring from the initial operation. Given the high prevalence and detrimental effect of level VI lymph node invasion, an adequate dissection of the level VI lymph nodes is of great importance.

The indications for prophylactic central neck dissection (pCND) remain controversial. The guidelines in Europe and America recommend that pCND should be performed primarily in patients with stage T3, T4, and cN1b carcinoma (11). In China and Japan, pCND is performed routinely, based on the rationale that 50% of occult cervical lymph node metastases are missed during preoperative examination but detected postoperatively, which in turn impacts staging and postoperative management (12). In particular, the guidelines in China emphasize parathyroid and RLN preservation, and concomitant ipsilateral pCND, to reduce the risk of postoperative complications and to improve quality of life (13).

Since the cervical esophagus is mainly located on the left, there is minimal lymphatic and connective tissue posterior to the left RLN. Conversely, on the corresponding area on the right, posterior to the RLN, anterior to the prevertebral fascia, and superficial to the esophagus, lymphatic and fatty tissue of the level VIb can be found. The morbidity of level VIb lymph node metastasis in patients with recurrence is reportedly significantly higher than the morbidity of patients who are undergoing surgery for the first time (12). In our study, patients with level VIb lymph node metastasis accounted for 13.7% of the total cohort (14/102) (14), which is consistent with the range reported in the literature (7.0% to 27.03%) (15). However, level VIb lymph node dissection is complex and may induce several complications. An inadequate dissection will enhance operative difficulties, increase the risk of injury to the RLN and parathyroid glands during surgery, and delay the opportunity for radical excision (16). It is therefore crucial to develop an efficient and thorough technique for level VIb lymph node dissection.

In conventional level VI dissection, especially dissection in the level VIb area, the middle and lower two-thirds of the right RLN are routinely mobilized circumferentially (17), and the medial and lateral lymphatic connective tissue around the RLN are dissected (18). Excessive mobilization of the RLN may increase the risk of injury to it, by way of physical injury from repeated traction (19), *via* electrical injury by electric currents during the severing of the tracheoesophageal branches (20), or by neural degeneration and necrosis by devascularization (5).

As for the number of dissected lymph nodes, our study compared the F-R-RLN dissection with the conventional dissection technique and found that there were no significant differences between the experimental and control groups in terms of the number of dissected lymph nodes in the level VIa and VIb compartments, or in the relative incidence of metastasis. In the experimental group, the median number of dissected level VIa lymph nodes was 6 and the median number of dissected level VIb lymph nodes was 2.5, which is in accordance with the literature (17). The number of level VI lymph nodes dissected *en bloc* in the experimental group was significantly greater than the number of lymph nodes obtained from simple level VIa dissection (Z-score = -3.080, *p* = 0.002), which implies that F-R-RLN dissection can achieve thorough level VIb dissection.

Apart from the complete dissection of lymphatic connective tissue, some other benefits might be associated with F-R-RLN dissection, as well. Firstly, injury to the RLN caused by repeated traction is avoided by slightly sparing the RLN laterally. Secondly, the RLN branches are preserved as much as possible, with only the medial tracheoesophageal branch sacrificed. Thirdly, the blood supply is preserved by maintaining a thin layer of connective tissue between the RLN and common carotid artery, which encases the vasculature (**Figure 3**). The incidence of transient vocal cord dysfunction after level VIb lymph node dissection was 7.4% according to the literature (6). The incidence in the experimental group in our study was 1.7% (1/60), and it was completely resolved 1 month after surgery. A possible explanation is that an esophageal branch of the RLN was accidentally severed by the bipolar electric scalpel 3 mm away from the RLN trunk during level VIb dissection. The degree of signal amplitude loss as quantified by R2p/R2d in the experimental group was significantly lower compared to the degree of signal amplitude loss in the control group. The ratio exceeded 90% in 63.3% of patients in the experimental group, which represented a significant improvement compared with the results from patients undergoing the traditional dissection technique (35.7%). According to the literature data (21), there is a close correlation between an intraoperative decrease in the RLN electrical signal amplitude and postoperative RLN paralysis (22). The results of our study show that F-R-RLN dissection helps to protect the RLN and mitigate the risk of nerve signal interruptions. However, there was no significant difference between the two groups in the incidence of transient vocal cord dysfunction. There was just one case of vocal cord dysfunction in each group. It is difficult to interpret this finding due to the low sample size and the fact that our surgeons were highly skilled. A larger sample size may be needed to substantiate our findings.

Regarding the protection offered to the parathyroid gland, this was initiated prior to the dissection. If the parathyroids were in their usual site, the parathyroid glands and the pedicle of the blood vessels were preserved *in situ* (**Figure 4**). However, if the parathyroid glands could not be clearly exposed, the sternothyroid ligament was still raised because there were no lymph nodes evident in the ligament, representing vestigial tissue from embryogenesis (23). In some cases, the parathyroid glands could be detected after elevating the ligament. The glands were preserved in the same way as described above. It has been reported that the incidence of transient and permanent hypoparathyroidism ranges from 14 to 60% and 4 to 11%, respectively (24). The incidence of unintentional parathyroidectomy ranges from 3.7% to 29.0% in the literature (25). In our study, the preservation rate of the parathyroid glands was 76.6% (46/60), and the incidence of unintentional parathyroidectomy was 6.7%, which is in line with previous reports. For patients undergoing total thyroidectomy in the experimental group, the incidence of postoperative transient hypoparathyroidism was 28.1% (9/32), and no permanent hypoparathyroidism occurred. There were no significant differences between the experimental and control groups in the rates of *in situ* preservation, transplantation, detection, or hypoparathyroidism, which suggests that F-R-RLN dissection is an effective method for parathyroid preservation.

The chylous leakage usually occurs after left level IV dissection and seldom happens after left or right level VI dissection. In our research, all patients underwent right level VI dissection and there were no cases of chylous leakage. Chylous leakage should be noted as a possible complication after level VI lymph node dissection due to aberrant drainage.

## Conclusion

Monitor Guided Fang’s Capillary Fascia Preservation Right RLN Dissection Technique offers some advantages over conventional techniques, including more thorough exposure and dissection of the level VI lymph nodes while preserving the RLN. This novel method can avoid excessive dissection of the RLN, reduce the probability of thermal injuries, and protect the blood supply to the RLN, which might decrease the incidence of postoperative complications and might improve surgical safety outcomes.

## Data availability statement

The original contributions presented in the study are included in the article/**Supplementary Material**. Further inquiries can be directed to the corresponding author.

## Ethics statement

The studies involving human participants were reviewed and approved by The ethics review board of the Beijing Tongren Hospital, Capital Medical University. The patients/participants provided their written informed consent to participate in this study.

## Author contributions

QS, JQX, and JGF contributed to the conception and design of the study. QS designed the experiment and organized the database. JQX wrote the first draft of the manuscript. QZ, XC, LZH, HZM, LF, HSZ, ML and RW contributed to the statistical analysis. All authors contributed to manuscript revision and have read and approved the final submitted version.

## Funding

This study was financially supported by the Beijing Natural Science Foundation Program and the Scientific Research Key Program of the Beijing Municipal Commission of Education (no. KZ201910025034), the Beijing Municipal Administration of Hospitals’ Ascent Plan (no. DFL20180202), the Capital Health Development Research Project (no. Shoufa-2018-2-2054), the National Key Research and Development Plan (2020YFB1312805), and the China Health Promotion Foundation and Thyroid Research Project for Young and Middle-Aged Doctors.

## Conflict of interest

The authors declare that the research was conducted in the absence of any commercial or financial relationships that could be construed as a potential conflict of interest.

## Publisher’s note

All claims expressed in this article are solely those of the authors and do not necessarily represent those of their affiliated organizations, or those of the publisher, the editors and the reviewers. Any product that may be evaluated in this article, or claim that may be made by its manufacturer, is not guaranteed or endorsed by the publisher.
